# Childhood epilepsies: What should a pediatrician know? 

**DOI:** 10.17712/nsj.2017.1.20160244

**Published:** 2017-01

**Authors:** Fahad A. Bashiri

**Affiliations:** *From the Division of Pediatric Neurology, Department of Pediatrics, College of Medicine, King Saud University Medical City, King Saud University, Riyadh, Kingdom of Saudi Arabia*

## Abstract

Seizures in children are among the most common neurological disorders. A pediatrician should know how to approach a child who presents with a seizure. This review will focus on points that are important in the evaluation of children who have experienced seizures. A comprehensive and neurologically focused framework for history taking and a thorough clinical examination are the cornerstones in diagnosing and managing seizures. This article reviews the clinical approach to the diagnosis, investigation, and management of epilepsy in children, excluding neonatal seizures. A pediatrician should also be aware of common epilepsy syndromes that occur in children such as Benign Childhood Epilepsy with Centro-Temporal Spikes, and childhood absence epilepsy.

Seizures are common with a prevalence of 0.5-1% in the general population. The incidence of epilepsy in children ranges between 41-187/100,000.^1^ Each year, approximately 150,000 children and adolescents experience their first unprovoked seizure, and approximately 30,000 of them are diagnosed with epilepsy.^1^ A recent study estimated that the overall prevalence estimate for 2012 was 6.8 per 1,000 children, and the incidence estimate for 2012 was 104 per 100,000 of the pediatric population.^2^ A seizure is defined as a transient alteration of consciousness, manifested as a specific behavioral and motor activity due to excessive electrical discharges from a group of cerebral neurons.^3^ Epilepsy is a disorder of the brain characterized by an enduring predisposition to generate epileptic seizures, and it requires the occurrence of at least one epileptic seizure.^4^ Pediatricians get scared and will not feel comfortable to deal with children who have epilepsy. Pediatricians should be aware of this disorder and should know how to approach a child who presents with a seizure. The objective of presenting this review is to shed some light on the important points of the evaluation of children presented with an epileptic seizure.

## History taking

History taking is very crucial in the evaluation of a child who presented with a seizure. The physician should ask the parents or the witness about the event. The physician should listen initially to the detailed description of the entire episode. To have a systematic approach, the event can be divided into 3 stages: before, during, and after the event.

## Before the event

History should cover the following questions: Was there any warning or aura? Auras consist of subjective symptoms that usually occur at the beginning of a seizure. They can manifest as somatosensory auras like paresthesia, visual or auditory hallucinations or illusions, olfactory auras in the form of abnormal smell perception, gustatory auras as in abnormal taste perception, autonomic ones like hot flushes, abdominal auras, or psychic ones.^4^ The aura usually represents a focal-onset seizure. Patients with temporal lobe epilepsy can have epigastric pain, and déjà vu, which is the phenomenon of having a strong sensation that an event currently being experienced has been experienced in the past. Paresthesias can be localized to the parietal lobe, and visual distortions may be localized to occipital lobe epilepsy.^5^,^6^ Generalized epilepsy is not associated with auras, and if it is reported or suspected at the onset of the seizure, the patient has to be re-evaluated for possible focal onset seizure.

Other questions that need to be looked into are: What was the child doing? Was he sleeping, awake, or even talking? Was there any loss of consciousness? Was there any history of fever or any illness such as flu-like symptoms, vomiting, or diarrhea? Were there any triggers for this spell?

## During the event

It is essential to obtain detailed information about the event from a witness and the following questions must be asked: Which side of the body did the seizure start in? Did it start on one side, and was there any head or eye deviation to one side? Was consciousness impaired? Did the child remember anything? Was the patient able to talk? Were there any repetitive movements such as lip smacking, chewing, swallowing, or finger rubbing? This symptom will indicate automatism. Was there any change in the skin color or any oral cyanosis? Was there any tongue biting or loss of bladder or stool control? Was there any dystonic posturing of the limb? How long did the spell last?

## After the event

Regarding the period immediately after the event (postictal period), the following questions should be answered: How did the patient feel? Was there any confusion or restlessness? Was there any postictal sleepiness that commonly happens after generalized epilepsy attacks? How long did this period take to go back to baseline? Was there any body weakness that is suggestive of a focal-onset seizure?

## Other questions to consider

If the patient is known to have epilepsy, ask about seizure control, or any change in seizure semiology. How is the compliance with antiepileptic drugs? Was there any history of trauma, previous fever, or febrile seizure? Was there any precipitating factor such as sleep-deprivation?

Past medical history including pregnancy and birth history must be investigated. Whether there was difficulty during labor, any admission to the neonatal care unit, any neonatal seizure, any history of central nervous system infection, such as meningitis or encephalitis, any history of tumor or cerebrovascular accident, or any history of traumatic brain injury.

Developmental history is also essential in patients with a seizure. All domains of the developmental milestones should be covered in the history taking. If the child is attending school, detailed evaluation about school performance and any history of attention problems should be clarified. It is well known that patients with epilepsy have comorbidities such as attention-deficit hyperactivity disorders, anxiety disorders, and learning difficulties. Population-based studies showed that children with epilepsy are at high risk for behavioral and mental health problems,^7^ so it is crucial to ask about individual school performance and any evidence of attention disorders or hyperactivity.

## Family history

It is well known that some of the epileptic syndromes are inherited,^8^ so physicians have to ask if there is any family history of epilepsy, developmental delays, or neuropsychiatric disorders.^9^ Social history is imperative as well, and the physician has to ask about the level of the parents’ education; this is vital while counseling and educating the family about the condition.

## Allergy and medications

The physician should ask about history of allergies, especially to antiepileptic drugs. A skin rash is one of the manifestations of such an allergy. However, it should be considered cautiously in the pediatric age group because it can be a secondary symptom of viral infection rather than a drug allergy. The physician should also ask about the patient’s medications in details including the dosage, duration of therapy, compliance, recent medication changes, and any side effects of the given medicines.

Video recording the event by parents is an excellent tool for giving an even more detailed idea about the event and should be utilized, especially in today’s age with the availability of electronic devices with high-quality cameras.

## Physical examination

All children who present with a history of suspected seizure should have thorough physical and neurological examinations. The physician should pay attention to the Vital signs, including temperature. The growth parameters: weight, height, and head circumference should be plotted on the parameter chart. The physician should look for any dysmorphic feature; it is well known that some of the syndromes associated with epilepsy have specific dysmorphic features.^9^,^10^ Signs of trauma or bruises may indicate brain injury. The skin should be examined for stigmata of neurocutaneous syndromes such as hypopigmentation or hyperpigmentation, port-wine staining, and skin hemangiomas. Fundal examination for papilledema, optic atrophy, or hyperpigmented retina. Asymmetry in the size of the limbs may indicate perinatal cerebral insult and symptomatic seizure. The full neurological examination includes studying the mental status, cranial nerves, motor & sensory nerves, reflexes, coordination, and gait. Abdominal examination should be performed, specifically looking for organomegaly.

After finishing the history and examination, the physician should formulate differential diagnosis about the case and classify the seizure.

## Diagnostic approach

Laboratory testing should be guided by clinical presentation of the child. Serum electrolytes such as glucose, calcium, magnesium, and sodium are essential.^11^ White blood cell count should be considered in any child who presented with fever. If sepsis is not suspected clinically, blood culture is not indicated in a patient presenting with seizure or even status epilepticus.^11^ There is insufficient data to support the performance of cerebrospinal fluid (CSF) studies in children who presented with status epilepticus in the absence of clinical suspicion of Central Nervous System (CNS) infection.^11^ Metabolic and genetic testing is optional and may be considered when the initial evaluation reveals no etiology, especially if the history is suggestive of metabolic disorders.^11^ In our population, the overall rate of consanguinity in Saudi Arabia is estimated to be 52.3%.^12^ Because of this, inborn errors of metabolism tend to be more common than in western populations.^13^ Furthermore, screening for common treatable epileptic syndromes should be considered. Electroencephalography (EEG) study is not indicated as an emergency unless it is required to rule out non-convulsive status epilepticus or subclinical seizure. The EEG can be done as an outpatient and should be done during awake, drowsy, and sleep states. Epilepsy is a clinical diagnosis, and normal EEG does not rule out epilepsy. However, EEG is crucial in classifying epileptic syndromes, tailoring the choices for antiepileptic drugs (AED), and sometimes help in predicting the outcome.^11^ Neuroimaging is not indicated in the first unprovoked seizure as an emergency unless the child has a focal neurological deficit, or the activity does not return to baseline within several hours after a seizure.^11^ CT scan of brain should be carried out if there are signs of increased intracranial pressure, a history of trauma, a presence of a VP shunt, or bleeding disorders.^3^ Magnetic Resonance Imaging (MRI) of the brain is superior to CT scanning. The indication of MRI brain includes, any child under one year of age, any child with a focal-onset seizure, cognitive or motor impairments, any child who has an unexplained focal neurological deficit, and an EEG that does not have features of benign partial epilepsy or primary generalized epilepsy.^11^

The recommendation in the infancy-age group is different from the older-age group; video EEG recording is superior to routine EEG. Neuroimaging is recommended at all levels of care in any infant presenting with epilepsy.^14^

## Treatment. Acute management

The initial management of a child who presented with active seizure is to focus on stabilization of the child and to stop the seizure. Airway, breathing, and circulation is the priority. Withdraw blood for glucose, calcium, magnesium, and sodium. If the patient has any electrolyte disturbances, it should be corrected immediately. Glucose should not be given to the child if he is on a ketogenic diet because this will break the ketogenic state. Early treatment of status epilepticus (SE) is associated with early cessation of seizure and better outcome. On the other hand, late intervention is associated with worse outcome. Home management of SE with rectal diazepam or buccal midazolam resulted in shorter duration of the seizure.^15^

**Figure 1:**
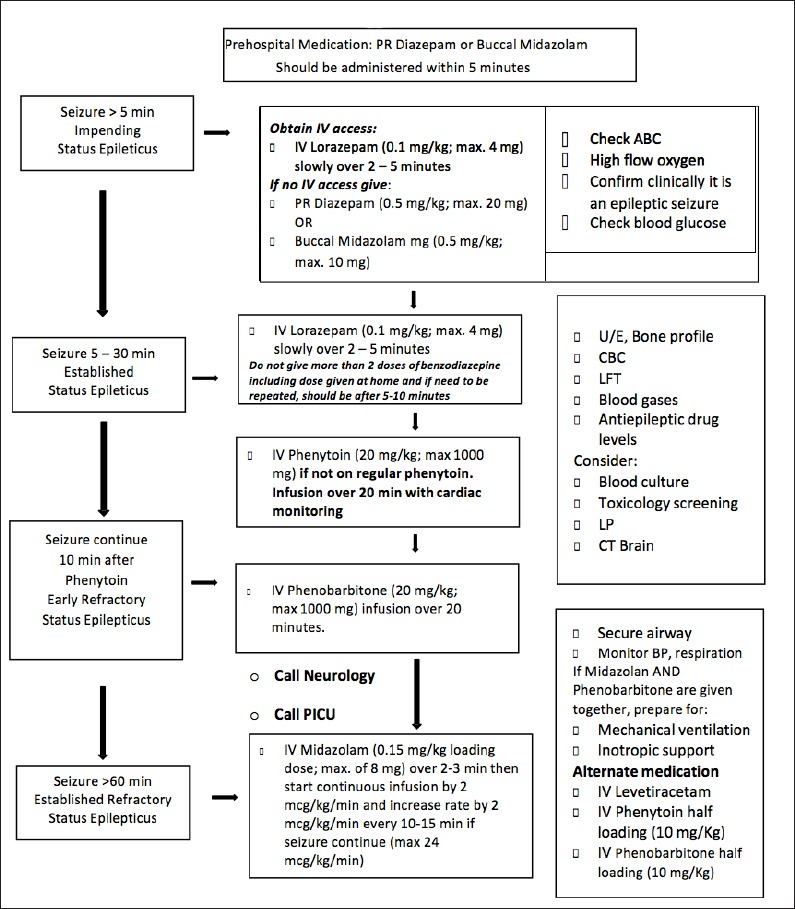
- Algorithm for acute management of status epilepticus in children. PICU - Pediatric Intensive Care Unit- IV - Intravenous, PR - per rectum, ABC - airway, breathing, and circulation, BP - blood pressure, CBC - complete blood count, LFT - liver function tests, LP - Lumbar Puncture, min - minimum, max - maximum

Benzodiazepine is the first-line therapy for SE. Phenytoin or fosphenytoin is the second-line therapy in many guidelines for status epilepticus.^11^ If the seizure is prolonged and does not stop even after the second-line therapy, the treating physician should use third-line therapy, which includes phenobarbitone. At this stage, the treating physician should be ready for mechanical ventilation and inotropic support, and should involve the intensive care unit and the neurology team. Other alternative medicines that can be considered are levetiracetam and valproic acid. In cases of refractory and super-refractory SE Intravenous (IV) midazolam infusion, IV pentobarbital, ketamine, and propofol should be considered. **Figure 1** shows the algorithm for acute management of status epilepticus.

## Chronic management

After stabilization of the child, the treating physician should decide about the choices of antiepileptic drugs, which will depend on multiple factors; the age of the child, the type of seizure, the risk of seizure recurrence, and other comorbidities or associated medical illnesses. There are general guidelines for initiating the treatments; monotherapy is better than multiple therapies, and starting with a small dose and increasing slowly till reaching seizure control is reached or any side effect develops is preferable. Parents should be advised about possible side effects of the medications. The physician should keep in mind that some of the AED can worsen seizures; carbamazepine, phenytoin, vigabatrin, and tiagabine may exacerbate CAE, Juvenile Absence Epilepsy (JAE), and Juvenile Myoclonic Epilepsy (JME). Dravet’s syndrome and JME can get worse with lamotrigine.^16^ **Table 1** Summarizes common antiepileptic drugs, indications, common side effects and maintenance doses.

**Table 1:** Common antiepileptic drugs, indications, common side effects and maintenance doses.

Drugs	Indications	Side effects	Maintenance (mg/kg/d)
Carbamazepine	Generalized tonic-clonic, partial, seizures	Rash, hepatitis, diplopia, aplastic anemia, leukopenia	10 – 40
Clonazepam	Myoclonic, akinetic, partial seizures, infantile spasms, Lennox-Gastaut	Fatigue, behavioral issues, salivation	0.05 – 0.30
Ethosuximide	Absence epilepsy	GI upset, weight gain, lethargy, SLE, rash	20 – 40
Felbamate	Refractory Severe epilepsy	Aplastic anemia, hepatotoxicity	15 – 45
Gabapentin	Partial and secondary generalized seizures	Fatigue, dizziness diarrhea, ataxia	20 – 70
Lamotrigine	Complex partial, atonic, myoclonic, absence, tonic-clonic, Lennox-Gastaut, infantile spasm	Headache, nausea, rash, diplopia, steven-Johnson syndrome, GI upset	5 – 15
Levetiracetam	Adjunctive therapy for refractory partial seizures	Headache, anorexia, fatigue, infection, behavioral issues	10 – 60
Oxcarbazepine	Adjunctive therapy for partial seizures	Fatigue, low sodium, nausea, ataxia, rash	10 – 45
Phenobarbital	Generalized tonic-clonic, partial myoclonic	Sedation, behavioral issues	2 – 6
Phenytoin	Generalized tonic-clonic, partial, atonic, myoclonic, neonatal	Gum hyperplasia, hirsutism, ataxia, steven-Johnson syndrome, lymphoma	4 – 8
Topiramate	Complex partial seizures	Fatigue, nephrolithiasis, ataxia, headache, tremor, GI upset	1 – 9
Valproic acid	Generalized tonic-clonic, absence, myoclonic, partial, akinetic, infantile spasm	GI upset, liver involvement, tremor, alopecia, sedation, weight gain	10 – 60
Vigabatrin	Infantile spasm, adjunctive therapy, for refractory seizures	Weight gain, behavior changes, visual field constriction	30 – 150
Zonisamide	Adjunctive therapy for focal seizures, atonic, infantile spasms	Fatigue, ataxia, anorexia, GI upset, headache, skin rash	2 – 8

SLE - systemic lupus erythematosus, GI - gastrointestinal

## Non-pharmacological therapy

Ketogenic diet, vagal nerve stimulation, and epilepsy surgery are well-known modalities of treatments in medically refractory epilepsy. However, a specialized epilepsy doctor should decide about the choices. That is usually carried out only in specialized centers with the facility of epilepsy monitoring units.

## Febrile seizures

Febrile seizure is not classified as an epileptic seizure. The general practitioner and pediatrician should know how to manage a child, and deal with the parents of a child with febrile seizure. Febrile seizures are the most common seizures in children from the age of 6 months to 5 years old. It occurs in approximately 3% of children in this age group. Approximately 2-5% of the children in the United States and Western Europe will have experienced at least one febrile seizure by the age of 5.^17^ Most of the febrile seizures are simple; single episode lasting less than 15 minutes and generalized. Complex febrile seizures usually have multiple recurrences in 24 hours, and can be focal and prolonged (more than 15 minutes).^17^ Neuroimaging and EEG are not indicated for simple febrile seizures.^18^ Lumbar puncture is not indicated in simple febrile seizures unless there are meningeal signs and concerns of a CNS infection.^18^ The recurrence rates range between 30% and 50%. The risk of occurrence of epilepsy in children with febrile seizures is 2-4%. It increases to 3.5 times if the seizure is a complex febrile seizure, and to 3.8 times if it occurs after 3 years of age.^19^ Positive family history of epilepsy will increase the risk by 7.3 times. If there are multiple seizure episodes and focal seizures, the risk increases by 10-11.7 in multiple seizures and 7.9-11.7 times in focal seizures.^19^ A simple febrile seizure is a benign condition and has an excellent prognosis. Long-term treatment with antiepileptic drugs is not needed. The risk of toxicities associated with antiepileptic drugs outweigh the relatively minor risks associated with simple febrile seizures.^20^ Parents should be counseled and reassured about the benign course of simple febrile seizures. Antipyretics will not prevent the occurrence of febrile seizures but may improve the child’s comfort.^20^ Rectal diazepam and buccal or nasal midazolam are effective in terminating the seizure.^20^

## Common epilepsies in childhood age group: Benign Childhood Epilepsy with Centro-Temporal Spikes (BECT)

The BECT is the most common idiopathic focal epileptic syndrome in childhood. Estimated prevalence is 20-25%. The age of onset ranges between 2 and 13 years.^21^ Seizures occur predominantly during sleep; characterized by hemifacial motor seizures and may be preceded by somatosensory symptoms involving the inner cheek, tongue, and lips, then involve the hand or both the hand and the leg on the side ipsilateral to the involved facial side.^21^ Secondary generalization occurs in 20-54%.^21^ EEG characterized by normal background with spikes having biphasic or triphasic appearance located in the descending part of the rolandic strip represented in the record at T3-C5 or T4-C6, and are referred to as centrotemporal spikes.^22^ Antiepileptic drug treatment of BECT may be unnecessary in most of the cases and is used only if seizures are frequent, prolonged or occurring during daytime.^23^ Recent studies showed that a significant percentage of children with BECT have neuropsychological problems such as attention and learning problems.^24^ This neuropsychological disturbance is related to frequent epileptiform discharges during non-rapid eye movement sleep, the age of onset of epilepsy, in addition to the side effects of antiepileptic drugs.^25^

## Childhood absence epilepsy

The CAE is the most common idiopathic generalized epilepsy in childhood, it accounts for 5-10% of childhood epilepsy. The age of onset commonly ranges between 5 and 7 years old. It is more common in girls. Seizures classically are absence seizures which are usually brief, and mild facial myoclonus is common.^26^ Generalized tonic-clonic seizures can occur in 30-40% of children, but usually at an older age.^26^ The EEG is characterized by a normal background with 3-HZ generalized spike-wave enhanced by hyperventilation and becomes more fragmented during sleep. Treatment of choice is ethosuximide, valproic acid, and lamotrigine.^27^ Prognosis, in general, is good with remission in two-thirds of the children with CAE within 2 years of treatment with medications.^26^ Neurobehavioral comorbidity such as attention and learning difficulties are common.^26^,^28^

In conclusion, epilepsy is a clinical diagnosis and the pediatrician should be aware of the importance of focused history and examination to approach the child and initiate the treatment of common epileptic syndromes in children before referring to a specialized physician.
